# Connecting a Connectome to Behavior: An Ensemble of Neuroanatomical Models of *C. elegans* Klinotaxis

**DOI:** 10.1371/journal.pcbi.1002890

**Published:** 2013-02-07

**Authors:** Eduardo J. Izquierdo, Randall D. Beer

**Affiliations:** Cognitive Science Program, Indiana University, Bloomington, Indiana, United States of America; Université Paris Descartes, Centre National de la Recherche Scientifique, France

## Abstract

Increased efforts in the assembly and analysis of connectome data are providing new insights into the principles underlying the connectivity of neural circuits. However, despite these considerable advances in connectomics, neuroanatomical data must be integrated with neurophysiological and behavioral data in order to obtain a complete picture of neural function. Due to its nearly complete wiring diagram and large behavioral repertoire, the nematode worm *Caenorhaditis elegans* is an ideal organism in which to explore in detail this link between neural connectivity and behavior. In this paper, we develop a neuroanatomically-grounded model of salt klinotaxis, a form of chemotaxis in which changes in orientation are directed towards the source through gradual continual adjustments. We identify a minimal klinotaxis circuit by systematically searching the *C. elegans* connectome for pathways linking chemosensory neurons to neck motor neurons, and prune the resulting network based on both experimental considerations and several simplifying assumptions. We then use an evolutionary algorithm to find possible values for the unknown electrophsyiological parameters in the network such that the behavioral performance of the entire model is optimized to match that of the animal. Multiple runs of the evolutionary algorithm produce an ensemble of such models. We analyze in some detail the mechanisms by which one of the best evolved circuits operates and characterize the similarities and differences between this mechanism and other solutions in the ensemble. Finally, we propose a series of experiments to determine which of these alternatives the worm may be using.

## Author Summary

Maps of the connections between neurons are being assembled for several organisms, including humans. But connectivity alone is insufficient for understanding the mechanisms of behavior. Nowhere is this more obvious than in the nematode *C. elegans*, where the nearly complete connectome has been available for over 25 years yet little is known about the neural basis of most of its behavior. Here we combine known neuroanatomical constraints from the *C. elegans* connectome with a simplified body and environment, and use optimization techniques to fill in the missing electrophysiological parameters in plausible ways so as to produce worm-like behavior. We focus on one spatial orientation behavior, where the reactions to sensory input depend on the worm's internal state at the time of the stimulus: salt klinotaxis. By exploring the possibilities for what is unknown in ways that are consistent with what is known, we generate an ensemble of hypotheses about the neural basis of this behavior. Studying the structure of this ensemble, we formulate new experiments that can distinguish between the various hypotheses. This methodology is likely to accelerate the discovery and understanding of the biological circuitry underlying the behavior of interest, before a complete electrophysiological characterization is available.

## Introduction

In recent years, connectomics – the assembly and analysis of comprehensive maps of neural connectivity – has been growing by leaps and bounds. Partial connectomes now exist for several organisms, including the nematode *C. elegans* [1], [2], the primate cerebral cortex of the macaque monkey [3], the cortico-thalamic system of the cat [4], and the mouse retina and primary visual cortex [5]. Recent efforts have increasingly been aimed at collecting data about the structural connectivity of the human brain at different levels of detail [6]–[9]. Furthermore, there have been several developments in high-throughput serial electron microscopy that continue to accelerate the rate and resolution of data collection [10], [11].

In addition to the experimental assembly of connectome data, there has also been a growing interest in studying the large-scale network properties of these connectomes using graph theory [12]–[15]. The focus of this analysis has been on the global properties of the full network, such as small-world, scale-free properties, common motifs, degree distributions, vertex degrees, generalized eccentricities, number of complete subgraphs, clustering structures, etc. [16]–[23]. The dynamical consequences of network structure, such as signal flow and propagation of neuronal activity in response to artificial sensory stimulation [24], has also begun to be examined. Thus, connectomics can provide important insights into the general organizational principles of nervous systems and their impact on neural activity.

However, despite these considerable advances in connectomics, connectivity alone is clearly insufficient to understand the neural basis of behavior. Although network structure can certainly constrain neural activity, it does not uniquely determine it. Connectivity data must be integrated with neurophysiological and behavioral data in order to obtain a complete picture of neural function [25]–[28]. In addition, connecting a connectome to behavior requires a much finer-grained analysis of connectivity than is usually done. In addition to calculating such global network properties as degree distributions and clustering coefficients, the specific interneurons and functional pathways that connect the relevant sensory neurons to the relevant motor neurons must be identified and the electrophysiological properties of those components and connections must be characterized.

The nematode worm *Caenorhaditis elegans* is an ideal organism in which to explore in detail the link between neural connectivity and behavior. *C. elegans* has been an important model system for biological research in a variety of fields including genomics, cell biology, developmental biology, and neuroscience [29]–[33]. Among its many experimental advantages are its short life cycle, compact genome, stereotypical development, ease of propagation, and simplicity of the neuromuscular system. The complete cell lineage, which is invariant between animals, has been established [29]. Most importantly for neuroscience, the *C. elegans* connectome for the hermaphrodite, comprising 302 neurons and over 7000 connections, is by far the most complete to date [1]. Yet, despite its relatively simple nervous system, *C. elegans* displays a large repertoire of behavior including locomotion, foraging, feeding, touch withdrawal, and taxes involving smell, taste, and temperature [32], [34], [35]. In addition, the worm exhibits more complex behaviors such as mating, social feeding, learning and memory [36]–[41]. A variety of techniques exist for characterizing and manipulating these behaviors, including automatic visual tracking [42]–[45] and the use of microfluidics to finely control the structure of artificial soil-like environments [46], [47].

Given the availability of a nearly complete data set on its connectome and the fact that many of its behaviors have been well characterized, the major remaining obstacle to detailed analyses of the neural basis of behavior in *C. elegans* is a neurophysiological one. Until recently, electrophysiological analysis in *C. elegans* has been difficult due to its small size and pressurized body. However, substantial progress is now being made using whole-cell patch-clamp techniques [48], calcium imaging [49], and optogenetics [50], and optical and electrophysiological recordings in *C. elegans* are becoming routine [51]–[54]. In addition, electrophysiological studies in the closely-related but larger nematode *Ascaris* can be used to make inferences about *C. elegans* electrophysiology [55], [56]. Unfortunately, we are still a long way from knowing even which synaptic connections in this nervous system are excitatory or inhibitory, let alone the magnitudes of such connections or their time courses. Indeed, the shortage of electrophysiological data has been the main reason that few neuroanatomically-grounded models of *C. elegans* behavior have been undertaken, despite the fact that its connectome has been known for over 25 years (e.g., [57], [58]).

In order to address the current lack of electrophysiological data to match the comprehensive connectome data for *C. elegans*, one can turn to stochastic optimization techniques such as evolutionary algorithms applied to brain-body-environment models of a behavior of interest [59]–[61]. In this approach, the model is constrained to known neuroanatomy and the unknown electrophysiological parameters are evolved such that the behavioral performance of the entire model is optimized to match that of the animal. Since different runs of the evolutionary algorithm can produce different solutions with nearly the same behavioral performance, the result of this process is not a unique model, but rather an ensemble of possible models [62]. Clusters of similar solutions can then be identified within this ensemble and representative members from each cluster can be analyzed in detail as to how the observed behavior arises from the interaction between the neuroanatomically-constrained evolved neural circuit and the model body and environment in which it is embedded. The insights gained from these analyses can then be used to design experiments that distinguish between the various possibilities, focusing experimental effort where it is most crucial. The results of such experiments can in turn be used to further constrain subsequent evolutionary optimizations.

To demonstrate the utility of this approach, we focus here on salt klinotaxis, a form of chemotaxis in *C. elegans*. Klinotaxis is defined as a strategy for moving up a gradient through gradual changes in orientation directed towards the source [63]. Salt chemotaxis [64] is one of the most studied spatial orientation behaviors in the nematode. Orientation to salt is important for *C. elegans* because the bacteria on which it feeds release salt into the surrounding medium as a natural part of their metabolism [35]. Salt chemotaxis also exhibits plasticity, both in the form of habituation to high salt concentrations [65] and taxis reversal after association of salt with an aversive stimulus [66], [67]. Moreover, the sensory neurons involved in chemotaxis have been identified [68]–[70]. The behavior itself has an interesting substructure, consisting of at least two distinct strategies: klinokinesis, and the more recently discovered klinotaxis. Klinokinesis is defined as a biased random walk [71], [72]. A number of models of klinokinesis have previously been constructed [73]–[76]. As an orientation behavior, klinotaxis fundamentally involves brain-body-environment interactions, since the salt distribution detected by chemosensory neurons drives the motion of the body, which in turn changes the perceived salt distribution. Klinotaxis is a particularly interesting spatial orientation behavior because (unlike klinokinesis) it exhibits state-dependence: the reactions to sensory input depend on the worm's internal state at the time of the stimulus.

In this paper, we construct a neuroanatomically-grounded model of *C. elegans* klinotaxis by building on a previous sensorimotor model that did not include interneuronal pathways [77]. First, we identify a minimal klinotaxis circuit by systematically searching the *C. elegans* connectome for pathways linking chemosensory neurons to the neck motor neurons responsible for steering and then pruning the resulting network based on both experimental considerations and several simplifying assumptions. We then run a large set of evolutionary searches for the electrophysiological parameters of this minimal circuit that optimize a measure of chemotactic performance. Although this measure does not specifically reward klinotaxis, we find that a significant fraction of these searches successfully produce klinotaxis in a way consistent with both the nematode and the previous model. Next, we analyze in some detail the mechanisms by which one of the best evolved circuits operates, providing insight into how the observed sensorimotor transformations are actually implemented interneuronally. We then enlarge our analysis to characterize the similarities and differences between this mechanism and other solutions observed in the ensemble. Finally, we propose a series of experiments that can be performed to determine which of these alternatives the worm itself may be using.

## Results

### Minimal klinotaxis network

In order to identify candidate klinotaxis networks, we mined the *C. elegans* connectome using the chemosensory neurons as the root set and the neck motor neurons as the target set. We began with the maximal network, connecting all chemosensory neurons to all neck motor neurons. We then constrained this network based on experimental evidence and simplifying assumptions until we arrived at a minimal but neuroanatomically-grounded klinotaxis network.

**Figure 1:**
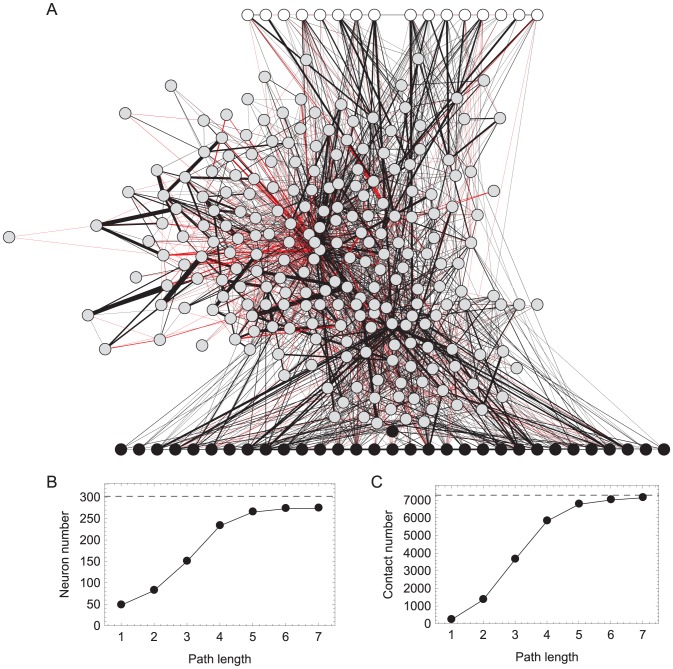
Searching the connectome for the klinotaxis network. **A**, Maximal network. All paths between chemosensory neurons (white disks) and neck motor neurons (black disks). Interneurons shown in gray. Chemical synapses shown as black lines. Directionality not shown. Gap junctions shown in red. Line thickness represents the relative number of chemical synapses or gap junctions between the two neurons. **B**, **C**, Number of neurons and number of chemical synapse and gap junctions in the network as a function of path length. Points represent the number of total neurons and contacts in the network. Dashed lines represent the total number of neurons and synapses and junctions in the connectome.

The maximal network contains all paths between chemosensory and neck motor neurons. The *C. elegans* chemosensory system enables it to detect a wide variety of volatile and water-soluble cues, with a total of 8 pairs of amphid neurons that are exposed directly to chemicals in the environment: ADF, ADL, ASE, ASG, ASH, ASI, ASJ, ASK [78], [79]. A total of 113 of the 302 *C. elegans* neurons are motor neurons [1]. As klinotaxis involves modulation of the side-to-side headswings, we were interested in the motor neurons that innervate the muscles in the head. We therefore only considered the 10 head and neck motor neuron classes: RIV, RIM, RMG, RMF, RMH, RMD, RME, SMB, SMD, and URA [80]. Figure 1A shows all of the paths between these two sets of neurons. Without additional constraints, the network connecting those two sets contains 90.72% of all neurons and 97.95% of all the chemical synapses and gap junctions in the connectome dataset.

The klinotaxis network is clearly contained within the maximal network, but is likely to involve a smaller subset of neurons. There are several ways to constrain the maximal network. One of the simplest and most effective is to limit the length of the paths because information is likely to be lost after traveling through many neurons due to nonlinearities and noise. From the maximal network we knew the longest path between chemosensory neurons and neck motor neurons was 7. As we limited the length of the paths, the size of the network (as measured by the number of neurons and chemical and electrical connections) was reduced dramatically (Figures 1B and 1C).

How do we decide what path length to constrain the network to? We considered a network to be *fully-connected* if signals from every sensory neuron could reach every motor neuron. Within the context of klinotaxis, this is an important criterion because it ensures that all information from the environment can be used to modulate motor neurons on both sides of the worm: dorsal and ventral. For any network, there is a minimal path length that meets the fully-connected requirement. For the network connecting all chemosensory neurons to all neck motor neurons that minimum was length 5, which included still 87.74% of all neurons and 92.98% of all the chemical synapses and gap junctions in the connectome dataset.

In order to further reduce the complexity of the network, we constrained the root and target set of neurons based on experimental results. The sensory neurons required for many chemosensory responses have been defined by killing identified neurons with a laser microbeam, and testing the operated animals for their behavioral capabilities. Studies have shown that chemotaxis to sodium and chloride ions are mediated mainly by the ASE sensory neurons [68]. Simultaneous ablation of all amphid and phasmid neurons except ASE spares chemotaxis, indicating that the role of ASE in water-soluble chemotaxis is unique [68]. There have been no studies in the motor neurons involved in the gradual turning observed during forward locomotion in klinotaxis. However, from studies of locomotion [81], we know SMB motor neurons set the amplitude of sinusoidal movement. Modulating the amplitude of sinusoidal movement at the timescale of head sweeps (see Methods) during forward locomotion can lead to gradual turning. This gradual turning is a likely candidate for producing the curvature in the translational direction of the worm (i.e., the direction of movement, see Methods).

**Figure 2:**
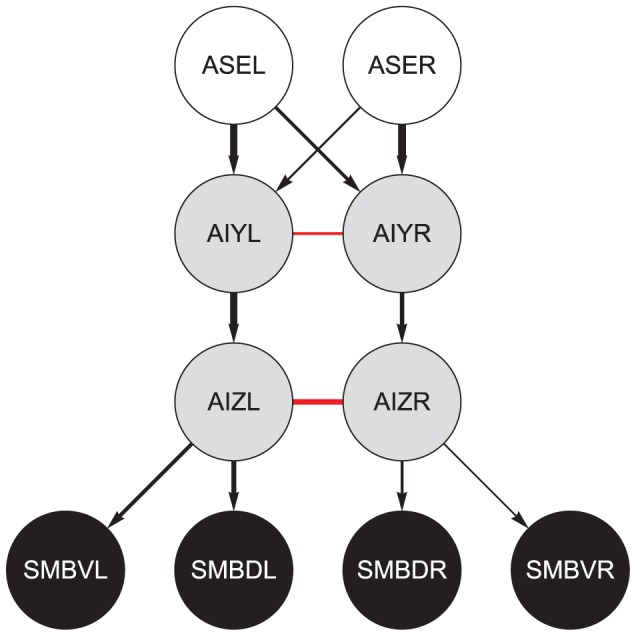
Neuroanatomy of the minimal klinotaxis circuit. White disks, chemosensory neurons; gray disks, interneurons; dark gray disks, motor neurons. Black connections represent chemical synapses. Red connections represent electrical gap junctions. The strength and polarity of the connections are still unknown. We show the strength and polarity from the best evolved network. *Arrowheads*, excitatory connections; *filled circles*, inhibitory connections. Line thickness shows relative connection strength. Color of the disk's border represents the sign of the bias term θ (*black*, positive; *gray*, negative). Thickness of the disk's border represents the magnitude of the bias. All parameters were symmetrical across the dorso-ventral midline.

The unconstrained network connecting ASE to SMB is still rather large, with 88.41% of all neurons and 93.76% of all chemical synapses and gap junctions in the connectome. We reduced this network to the minimal fully-connected one by once again constraining the path length. Constraining the network to paths of length 3 (the minimum consistent with the fully-connected criterion) reduces it to only 23 (7.61%) neurons and 276 (3.78%) chemical synapses and gap junctions. This allows us to test how much of the behavior can be accounted for by the most direct paths only, with indirect paths added in subsequent iterations of the model. There is, however, a further simplification that we can make to the network. Two neurons can be connected by one or more chemical and electrical synapses. We refer to the total number of chemical and electrical synapses between two neurons as the contact number. We simplified the network one step further by setting a threshold for the number of contacts between two neurons. The assumption is that the better-connected paths are more likely to have stronger interactions. When we constrained the network to paths with 2 or more contacts, we obtained a network that contained only two interneuron classes: AIY and AIZ (Figure 2). We refer to this network as the minimal klinotaxis network. Any further simplification breaks the fully-connected criterion.

The actual klinotaxis network falls between the two extreme networks: minimal (Figure 2) and maximal (Figure 1A). There are three main reasons why the minimal network is worth studying in more detail. First, the network is fully-connected: each of the sensory neurons can affect all of the motor neurons. Second, while the sensory neurons have been well identified, the circuits of interneurons that process sensory information are much less well characterized. The two interneurons that have been shown to be involved in klinotaxis, AIZ [63] and AIY [82], are included in the minimal network. This is important because it was not deliberately taken into consideration in the selection of the network; rather it emerged from the experimental constraints and simplifying assumptions. Finally, starting with the minimal network allows us to test the minimum neuroanatomy necessary to produce the behavior. As soon as the network fails in some respect and as more experimental data becomes available, the constraints can be relaxed and more components included in a systematic way.

### Population analysis

To identify neuroanatomically constrained neural networks for klinotaxis in *C. elegans*, we generated a population of 100 different networks using an evolutionary algorithm. Networks evolved reliably after 300 generations. Out of the 100 evolutionary runs, 17 failed to produce networks capable of efficient chemotaxis (chemotaxis index lower than 0.5, see Methods). Of the rest, we focused only on the highest-performing subpopulation, namely those networks having a chemotaxis index (CI) of at least 0.75 (*n* = 27). When tested over a longer assay, this subpopulation had an average CI of 0.87. All further analysis was limited to this high-performance subpopulation.

Networks were evolved in chemical gradients with conical shapes, where the chemical concentration falls as a linear function of the Euclidean distance to the peak. To test for generalization we measured chemotaxis index and reliability in a Gaussian-shaped chemical gradient (see Methods), which resembles the gradients used in laboratory tests of chemotaxis in *C. elegans* [64]. The measures in the Gaussian gradient closely matched those obtained in the conical gradient (Table 1). This experiment shows that evolved networks are not specialized for the shape of the gradient; instead, they embody a more general solution to the task of klinotaxis, making them appropriate for further study.

**Table 1:** Chemotaxis efficiency of the high-performance subpopulation in conical and Gaussian gradients.

	Gradient shape	
	Conical	Gaussian
Chemotaxis index (*CI*)	0.875±0.001	0.871±0.002
Reliability (%)	1.0±0.0	1.0±0.0

**Figure 3:**
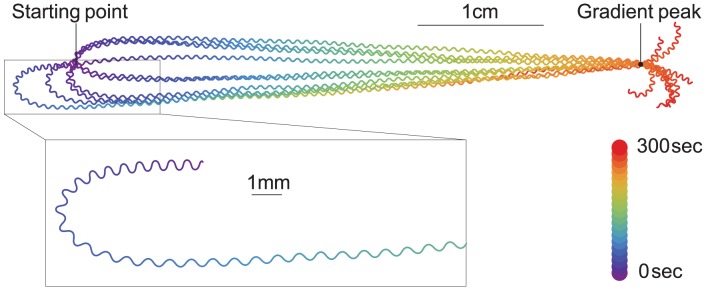
Behavioral trace. A representative model worm was placed at 10 random orientations 4.5 cm from the gradient peak and allowed to move for 300 sec. The gradient is Gaussian shaped. Trace color represents time. **Inset**: Enlargement of a gradual turn.

In order to determine the mechanism by which model worms reach the peak of the gradient, we first observed how the trajectories of virtual worms varied as a function of the model's random initial bearing (i.e., the angle difference between the direction of translational movement and the direction of the peak of the gradient) and then analyzed whether the trajectories met the two criteria for klinotaxis set out in previous work [77]. Figure 3 shows that model worms made a smooth turn until they were oriented in the direction of steepest ascent. Thus the output of the model was consistent with both real worm tracks during klinotaxis [63] and the previous theoretical model [77].

**Figure 4:**
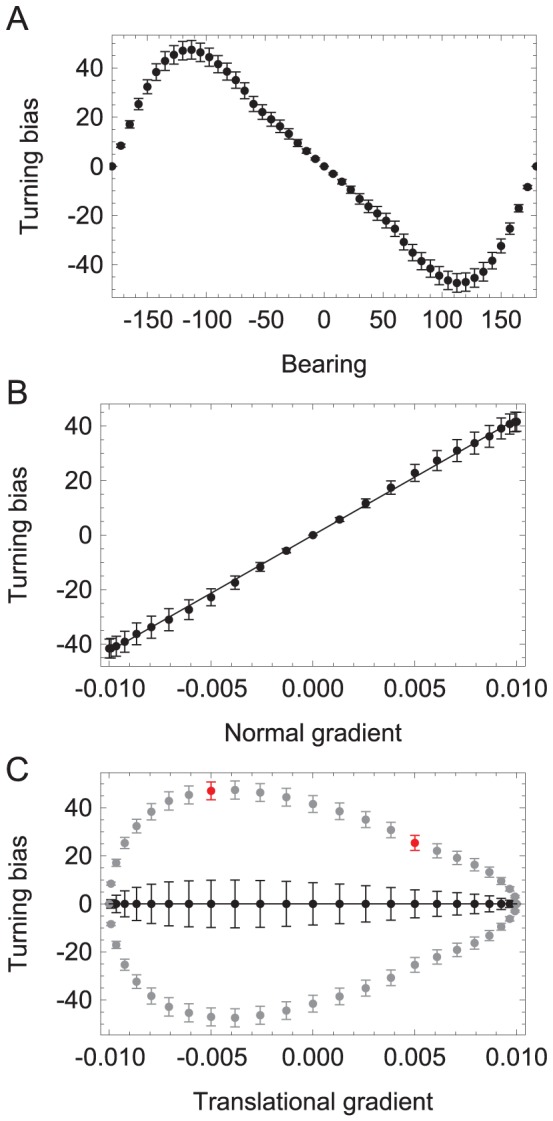
Klinotaxis analysis. **A**, Average turning bias vs. bearing. **B**, Average turning bias vs. the component of the gradient in the normal direction. **C**, Average turning bias vs. the component of the gradient in the translational direction. Black points represent all of the data averaged into bins according to the translational gradient. Gray points show the data separated between positive and negative turning bias. The two red points highlight the significance of the difference between the turning bias when moving down the gradient versus when moving up the gradient at the same angle. Error bars are standard error of the mean.

Klinotaxis has two necessary conditions: (C1) The organism continuously adjusts its orientation toward the line of steepest ascent; (C2) The adjustments in orientation are made on the basis of comparisons of the stimulus at a single point on the body as this point is swept from side to side over time. To ascertain whether networks met C1, we plotted track-curvature, quantified in terms of turning bias (see Methods), as a function of instantaneous bearing relative to the gradient peak (Figure 4A). According to C1, the algebraic sign of the turning bias should always be opposite to the sign of bearing. Figure 4A shows that this was indeed the case. To ascertain whether the optimized networks met C2, we plotted turning bias as a function of the amplitude of the gradient in the direction normal to the worm's translational movement (Figure 4B). This plot revealed that turning bias increased linearly with the amplitude of the gradient normal to the worm, as expected for a simple causal relationship between the concentration differences during head sweeps and turning bias. Furthermore, on average, turning bias was not affected by the model worm's movement in the translational direction (black points, Figure 4C). This finding suggests that turning bias in the model is controlled by changes in concentration sensed during the side-to-side head sweeps, as required by C2.

Each of the data points in the gradient in the translational direction (black points, Figure 4C) corresponds to the average over two distinct bearings. For example, +90 and −90 degrees both have 0 translational gradient. Their corresponding turning biases are nonzero, equal in magnitude but opposite in sign. So although the translational gradient does not influence the turning bias on average, when we studied the different cases more systematically we found some information in the translational direction: the magnitude of the turns were larger for negative translational gradients than for positive translational gradients (gray points, Figure 4C). There is a significant difference in the turning bias of the model worm when moving up the gradient at an angle than when moving down the gradient at that same angle (see red points, Figure 4C). Therefore, the magnitudes of the corrections are larger when the worm is heading away from the peak than when the worm is heading towards the peak. Although this is not a requirement of klinotaxis, it is an efficient component to the strategy exploited by the evolved model worms.

The sinusoidal relationship between turning bias and bearing, together with the linear relationship between turning bias and the normal component of the gradient, are qualitatively similar to the relationships observed in studies of klinotaxis in real worms [63]. This similarity is significant for two reasons. First, as we did not explicitly include selection criteria in the evolutionary algorithm to approximate these features, this similarity is an emergent property of the evolved networks. Second, the resemblance suggests that the model employs a klinotaxis strategy similar to the one used by real worms, making the model presented here especially appropriate for the generation of testable predictions concerning how the biological network functions.

### Sensorimotor transformation

**Figure 5:**
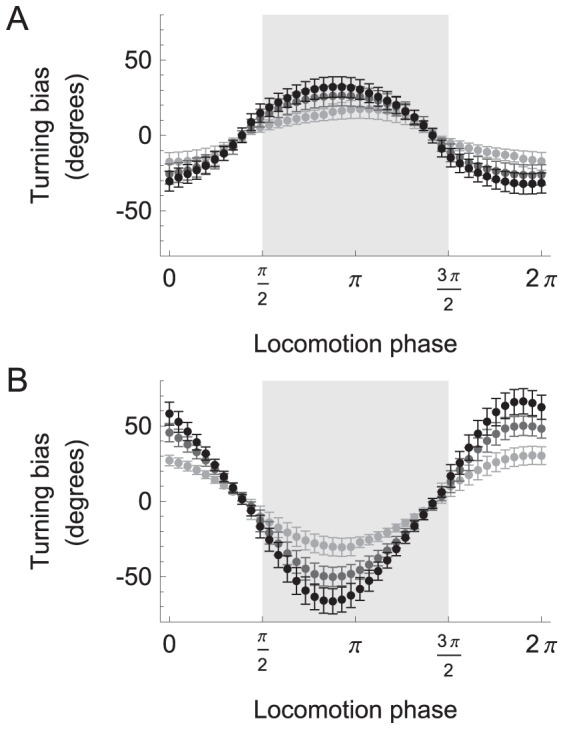
Phase sensitivity of orientation responses. **A**, Response to upsteps. **B**, Response to downsteps. Plots show turning bias vs. the phase of locomotion at which the concentration step occurred. Each point represents the average across all networks; error bars are standard error of the mean. Shades of gray indicate the magnitude of the concentration step (0.005 black, 0.00333 dark gray, and 0.00166 light gray). Positive and negative values of turning bias result, respectively, in counterclockwise and clockwise rotations of the translational vector. Abbreviations: VS, ventral head sweep; DS, dorsal head sweep (shaded region).

If we consider only the transformation that occurs between the sensors and motors, it is possible to compare the results of the neuroanatomically-grounded model with the previous simplified model. In order to do this, we studied how changes in concentration are transformed into changes in motor responses, as reflected by the worm's orientation, using single stepwise changes in concentration of different magnitudes at different points in the locomotion phase (increments in concentration: upsteps, Figure 5A; decrements in concentration: downsteps, Figure 5B).

Orientation responses were expressed in terms of turning bias, which was computed over a complete cycle of locomotion following the concentration step. We observed that turning bias varied as a sinusoidal function of the phase of locomotion at which the concentration change occurred (Figures 5A and 5B). This function had extrema near phases 0 and π, where the instantaneous velocity vector (*v*, see Methods) diverges most from the unbiased translational vector (*u*, the worm's direction of movement in the absence of external input, see Methods), and minima near phases of π/2 and 3π/2, where the instantaneous velocity vector diverges least from the unbiased translational vector. In the context of klinotaxis on a gradient, the instantaneous velocity vector at the time of an upstep signals the direction of the peak implied by such a step, whereas the instantaneous velocity vector at the time of a downstep signals the direction opposite to the peak. Thus, the model worm corrects its orientation relative to discrepancies between its velocity vector and the direction of the peak throughout the locomotion phase. The amplitude of the orientation response was proportional to the amplitude of the concentration step (Figure 5). This proportionality is important when the changes in concentration produced during dorsal and ventral sweeps have the same sign but different magnitudes.

The sensorimotor transformation in Figure 5 is qualitatively similar to the previous theoretical model [77], which suggests the principles of operation of the neuroanatomical network are consistent with the simpler model.

### Analysis of representative network

A neuroanatomically-grounded model allows us to go beyond overall sensorimotor transformations to examine the interneuronal implementation of klinotaxis. In this section we analyze in some detail one of the best evolved networks (Figure 2), a representative of many of the rest of the high-scoring subpopulation. The network's performance depends on the parameters that it evolved, but the solution is not brittle: there is a graceful degradation of the performance as parameters are independently perturbed away from the evolved values (see Figure S1).

**Figure 6:**
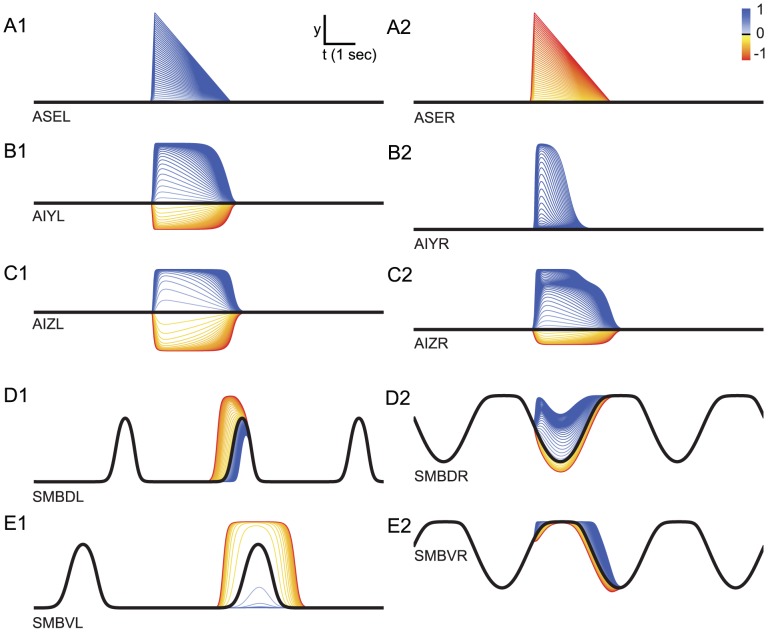
Neuron output traces in response to step changes in concentration in the best evolved network. **A**, Chemosensory neuron output, ASE. **B**, First layer interneuron output, AIY. **C**, Second layer interneuron output, AIZ. **D** and **E**, neck motor neuron output (**D**, SMBD; **E**, SMBV). Time is shown on the *x*-axis. Neuron output is shown on the *y*-axis. Each trace is color-coded according to the magnitude and sign of the step of the concentration step. The black trace represents the neuron output without input. The neuron output from left cells is shown on the left; the neuron output from right cells is shown on the right.

In order to understand how changes in concentration travel through the network, we studied how the synaptic potential (henceforth, output) of the neuron changed as a function of step changes in concentration of different sign and magnitude over the full spectrum that the model worms experience during klinotaxis runs (Figure 6). The dynamics of the chemosensory neurons follow directly from their definition (see Methods): ASER and ASEL react only to downsteps and upsteps in concentration, respectively (Figures 6A1 and 6A2).

The connections between the chemosensory cells ASEL and ASER and the first interneuron class (AIY) are excitatory and inhibitory, respectively (Figure 2). Therefore, upsteps in concentration move the membrane potential of both AIY cells upward and downsteps in concentration move the membrane potential of both AIY cells downward (henceforth, we will refer to the membrane potential as the activation of the neuron, with positive-going changes as increases in activation and negative-going changes as decreases in activation). How each AIY cell reacts to changes in concentration is a function of their bias parameter in relation to the strength and sign of the incoming chemical synapses from the chemosensory neurons. Because of the nonlinearity of the input-output relation (see Methods, Eq. 3), each AIY cell can only respond to changes in concentration within a certain range (henceforth, sensitivity). Also, given that the parameters of the network are not constrained to be left/right symmetric, the range of sensitivities of the two AIY cells can be different. In this network, AIYL is sensitive to small changes in concentration, positive or negative (Figure 6B1); whereas AIYR, due to a strong negative bias parameter (Figure 2), is only sensitive to large increases in concentration (Figure 6B2). Crucially, the ranges of sensitivities of the two cells are complementary, such that together they cover a broader range of the possible stimuli than individually.

The gap junction between cells drives the activation of the neurons closer together: the lower the resistance, the bigger the effect. The effect, however, is not always noticeable in output space due to the nonlinearity of the input-output relation: changes to the activation of the neuron can be masked by the saturation of the input-output relation. This is the case for the gap junction between the AIY cells in this network (Figures 6B1 and 6B2). Indeed, blocking the gap junction does not alter the dynamics of the output of the two interneurons significantly. From this experiment we conclude that the sensitivities of the AIY cells depend mainly on the incoming chemical synapses from ASE.

Neuroanatomically, AIZ cells only receive a chemical synapse from the AIY cell directly upstream. When we combine this with AIZ's own nonlinear response, we would expect the range of sensitivities to different steps in concentration to be a reduced set from AIY's. But this is not what we observe (Figure 6C). Unlike in the AIY interneurons, the gap junction between these AIZ cells plays an important role. The effect can be seen in AIZR best: even though AIYR is not sensitive to downsteps (Figure 6B2), AIZR is sensitive to them (Figure 6C2). This information is transferred not via the chemical synapses downstream, but via the lateral gap junction with AIZL. The AIZ gap junction plays a crucial role in redistributing and broadening the sensitivities to the changes in concentration between the left and right cells.

**Figure 7:**
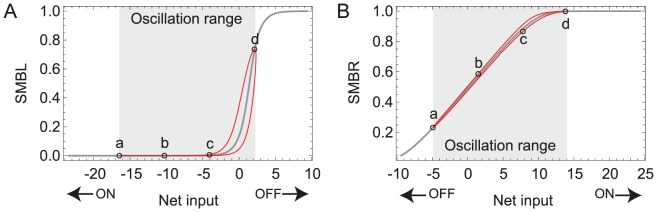
Dynamics of the neck motor neurons. Input-output diagrams for the left (**A**) and right (**B**) pair of dorsal and ventral motor neurons. Gray trace, steady-state input-output (SSIO) curve when the head sweep oscillation is absent. Red trace, instantaneous input-output relation when the head sweep oscillation is present. Arrows show the effects of output of the indicated chemosensory neuron on motor neuron input. Shaded areas show the range of oscillation. For each of the SSIO curves, there are dorsal and ventral motor neurons moving out of phase over the red trajectory due to the out of phase input from the oscillatory component. When the dorsal motor neuron is at point *a* in the curve, the ventral motor neuron is at point *d*, and vice versa. When the dorsal motor neuron is at point *b* in the curve, the ventral motor neuron is at point *c*, and vice versa. For the dorsal/ventral SMBL pair (**A**), an increase/decrease in chemical concentration would decrease/increase the output of the neuron in *d*, but not of the neuron in *a*; decreasing/increasing the difference between the two left neck motor neurons. For the dorsal/ventral SMBR pair (**B**), *a* and *d* represent the opposite regions: a neuron at *a* is more sensitive to changes in input than the other neuron at *d*; but the result is the same: an increase/decrease in chemical concentration decreases/increases the difference between the two neck right motor neurons. To different degrees, the same is the case for other points along the curve.

Unlike interneurons, motor neurons additionally receive an oscillatory input. Therefore, how they react to step changes in concentration depends on the phase of the locomotion cycle (Figure 6D–E) in which a change occurs. In order to understand their operation, we first consider their dynamics in the absence of sensory input. Figure 7 shows the steady-state input-output (SSIO) curve of the left and right motor neurons. The oscillatory input drives the motor neurons around the red trajectory, with dorsal and ventral cells out of phase. The key feature of the motor neurons is that the sensitive part of the SSIO curve is such that when one of the dorsal motor neuron is in the sensitive area of the curve, the ventral one is not, and vice versa. Even though the SSIO curves for the left (Figure 7A) and right (Figure 7B) pair of motor neurons are different, they share the same principles: (a) the sensitive region is biased with respect to the oscillatory range, and (b) increases in concentration move the input towards the most saturated part of the range; decreases in concentration move the input towards the sensitive part of the range. This is consistent with the principles observed in the motor neurons of the previous sensorimotor-only model [77].

It is the asymmetry in the location of the sensitive area in the SSIO curves of the motor neurons that allows for state-dependence in the network (Figure 7). We analyze first the left pair of motor neurons. When the concentration increases, AIZL's activation also increases, and for some range of magnitudes the neuron output increases as well (blue traces Figure 6C1). Given the inhibitory connection to the motor neurons (Figure 2), SMBDL and SMBVL receive less input as a consequence. As the dorsal and ventral neurons are out of phase, one is in the sensitive region of its SSIO curve and the other is not. Therefore, the output of one of the motor neurons decreases and the other one stays the same (compare blue trace in Figure 6D1 to Figure 6E1). This decreases the difference between the output of the dorsal and ventral motor neurons, which ultimately decreases the worm's turning. When the concentration decreases, AIZL's activation also decreases (red traces, Figure 6C1), and for some range of these changes in concentration the neuron output decreases as well. In this case, the motor neurons receive more input, and as a consequence of the bias in sensitivity, the output of one of the motor neurons increases and the other stays saturated (compare red trace in Figure 6D1 to Figure 6E1). This increases the difference between the output of the dorsal and ventral motor neurons, which ultimately increases the worm's turning.

Despite the differences in evolved parameter values, the effect is similar in the right motor neurons (Figures 6D2 and 6E2). When the concentration increases, AIZL's activation also increases, and for some range of magnitudes the neuron output increases as well (blue traces, Figure 6C2). Given the excitatory connection to the motor neurons (Figure 2), SMBDR and SMBVR receive more input as a consequence. As the dorsal and ventral neurons are out of phase, one is in the sensitive region and the other is not. Therefore, the output of one of the motor neurons increases and the other one stays the same (compare blue trace in Figure 6D2 to Figure 6E2). This decreases the difference between the output of the dorsal and ventral motor neurons, which ultimately decreases the worm's turning. When the concentration decreases, AIZR's activation also decreases (red traces, Figure 6C2), and for some range of these changes in concentration the neuron output decreases as well. In this case, the motor neurons receive less input, and as a consequence of the bias in sensitivity, the output of one of the motor neurons decreases and the other stays saturated (compare red trace in Figure 6D2 to Figure 6F2). This increases the difference between the output of the dorsal and ventral motor neurons, which ultimately increases the worm's turning.

**Figure 8:**
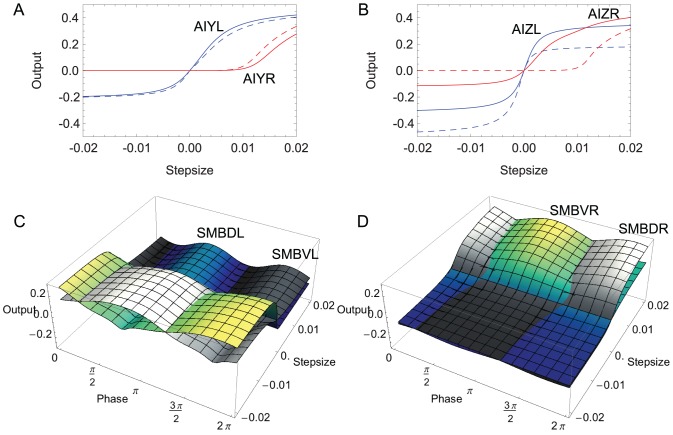
Range of sensitivity to changes in concentration. **A**, Interneuron class AIY. **B**, Interneuron class AIZ. Left neuron shown in blue; right neurons shown in red. Flat regions of the curve correspond to areas of no-sensitivity to input over that region. The slope of the curve denotes the degree of sensitivity to changes around that region of the input. For example, AIYR is sensitive to large positive changes in concentration only, whereas AIYL is sensitive to changes in concentration around the midline. Dashed lines show the sensitivity when the gap junction between those two neurons is blocked. In AIY, blocking the gap junction does not affect the sensitivity of its left and right neurons. In AIZ, blocking the gap junction results in substantial differences in the range of sensitivity. **C**, **D**, Sensitivity in the left and right pair of motor neurons as a function of the phase of locomotion, respectively. The dorsal motor neuron is shown in blue-green-yellow shades; the ventral motor neuron is shown in gray scale. Similar to the interneurons, the motor neurons have a selective range of sensory input over which they are sensitive. Unlike the interneurons, their sensitivity changes as a function of the worm's sinusoidal movement. Because of the out of phase oscillation between dorsal and ventral motor neurons, which neuron is more sensitive to a certain input swaps back and forth between them.

In order to understand the range over which each neuron is sensitive to changes in concentration, we visualized the difference in output between the no input and input conditions as a function of the magnitude and polarity of stepwise changes in concentration (Figure 8). In AIY, the sensitive regions of the neuron output for the left and right cells are different (Figure 8A): AIYL is most sensitive to small negative and positive steps whereas AIYR is most sensitive to larger positive steps. We can also use this analysis to study the role of the gap junction, by blocking it and comparing the changes in sensitivity to the unblocked condition. We observed almost no change in the sensitivities of the left and right cells when the gap junction is blocked (dashed curves, Figure 8A). In contrast, for AIZ, the sensitive regions of the neuron output for both the left and right are similar (Figure 8B): both are sensitive to small upsteps and downsteps, though AIZL is still most sensitive to small negative and positive steps and AIZR is biased towards larger positive steps. The difference in the range of sensitivity for the AIZ cells when the gap junction is blocked is dramatic (dashed curves, Figure 8B).

The surfaces in Figures 8C and 8D allow us to visualize how the sensitivities change as a function of locomotion phase, in addition to the magnitude and polarity of the step change in concentration. As for the interneurons, the main differences between the left and right pair of motor neurons is in the range over which they are sensitive to changes in concentration. The left pair of motor neurons is most sensitive to small downsteps and upsteps (Figure 8C), whereas the right pair is more sensitive to large upsteps (Figure 8D). This difference stems from the combination of sensitivities of the AIZ cells upstream (Figure 8B) and the dynamics of the motor neuron (Figure 7). The dorsal and ventral, left and right motor neurons add together to affect the dorsal and ventral muscles, respectively. Therefore, the different ranges of sensitivity are ultimately combined at the level of the dorsal and ventral muscles.

**Figure 9:**
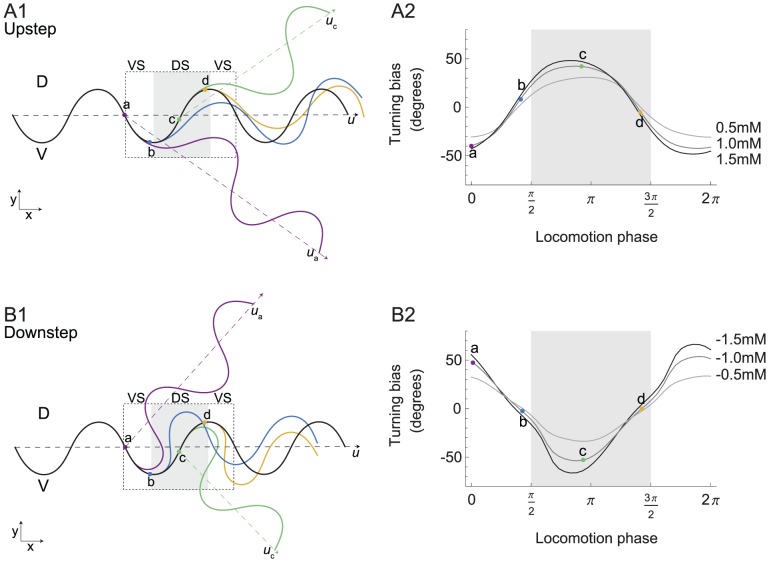
Orientation responses elicited by stepwise changes in concentration. **A**, Response to upsteps. **B**, Response to downsteps. **1**, Behavioral traces for four points in the locomotion phase. The black trace shows the trajectory in the absence of a concentration step. The colored traces show the trajectories obtained in response to concentration steps placed at the locations indicated by dots of matching color. The four locations chosen cover a complete cycle of locomotion (dashed box). Vectors indicating the direction of translation (dashed lines) are shown for no concentration step (*u*), and steps at locations *a* and *c* (*u_a_*, and *u_c_*). Abbreviations: DS, dorsal head sweep; VS, ventral head sweep (shaded region). **2**, Generalization of the turning bias as a function of the timing within the locomotion phase where the step is introduced. The example traces from **A1** and **B1** are represented by labeled disks in **A2** and **B2**, respectively.

The final issue in analyzing the mechanism of klinotaxis in this model circuit is to consider the behavioral effects of motor activity on the orientation of the body. How do the step changes in concentration result in changes in the translational direction? (cf. Figure 5). In order to answer this question, we analyzed the orientation responses produced by single stepwise changes in concentration (upsteps, Figure 9A; downsteps, Figure 9B).

Upsteps activate the ON cell, which reduces turning angle. The effect of the turning angle reduction on the worm's translational direction is dependent on the phase of locomotion. We consider two representative phases. First, an upstep at the midpoint of a ventral/dorsal head sweep (Figure 9A1, points *a/c*): turning angle is decreased for approximately the duration of a head sweep (2.1 secs, cf. Fig. 6). This persistent reduction in turning angle attenuates the ensuing dorsal/ventral turn; with the result that the worm's translational velocity vector rotates ventrally/dorsally (red/green trajectory, vector *u_a_*/*u_c_* vs. *u*). Both the dorsal rotation at point *a* and the ventral rotation at point *c* are appropriate orientation responses because the model worm turns in the direction of its instantaneous velocity vector at the time of the increase in concentration. Second, an upstep at the ventral/dorsal maximum (point *b/d*): no rotation occurs because the decrease in turning angle attenuates parts of the dorsal and ventral turns almost equally (blue/yellow trajectory). The absence of rotation at points *b* and *d* is appropriate because the model worm's instantaneous velocity vector at the time of the increase in concentration was the same as its translational direction. Downsteps activate the OFF cell, which increases turning angle. The analysis is the same as with upsteps, except that the turning increases instead (Figure 9B1). Crucially, the ventral rotation at point *a* and the dorsal rotation at point *c* are appropriate orientation responses because the model worm turns away from the direction of its instantaneous velocity vector at the time of the decrease in concentration (red/green trajectory, vector *u_a_*/*u_c_* vs. *u*).

To obtain a more complete understanding of how the simple rules for predicting changes in turning angle lead to correct orientation responses, we expanded the analysis of Figures 9A1 and 9B1 to include steps in concentration not only at points *a*–*d*, but also at the points in between (upsteps, Figure 9A2; downsteps Figure 9B2; cf. Figure 5).

This mechanism depends on three basic principles. (1) The two motor neurons are biased such that when one motor neuron is sensitive to sensory input, the other is not. (2) The signs of connections from sensory neurons to motor neurons are adjusted with respect to motor neuron bias such that ON cell activation reduces the curvature of the worm's path, whereas OFF cell activation increases the curvature of the worm's path. (3) The dynamics of sensory responses are adjusted so that changes in curvature are transient, lasting for approximately the duration of a head sweep. As a result, changes in curvature cause the worm's path to veer toward concentration increases and away from concentration decreases, unless the worm's head is moving in the direction of the gradient peak at the time the concentration change is encountered. These principles are similar to those found in the previous sensorimotor-only model [77]. The novelty of the analysis here lies in the implementation of the mechanism at the interneuronal level. The interneurons on the left and right side of the network show a certain amount of shared information about the changes in concentration, but also some degree of specialization: some changes in concentration are sensed by the left side of the network only and some changes in concentration are sensed by the right side of the network only. This feature allows the network to extend the coverage of the range of possible changes in concentration. Finally, the gap junctions between the interneurons can play a key role in distributing the sensitivities.

### Generalizations

The result of evolutionary optimization is not a unique model, but rather an ensemble of possible models. Given the underconstrained nature of optimization (due to the lack of electrophysiological data and the possibility of variability in both the available experimental data and behavior across individuals), understanding the structure of this ensemble is a key aspect of the approach we have taken in this paper. Studying an ensemble of models with different underlying parameters and similar behaviors provides opportunities to explore different possible mechanisms that could be operating in the worm. How does the mechanism that evolved in the network analyzed generalize to the rest of the high-scoring population? We examined the similarities and differences between, on the one hand, the evolved electrophysiological parameters and the interneuronal dynamics of the best evolved network and, on the other hand, the rest of the high-scoring subpopulation.

#### Interneuron types

**Figure 10:**
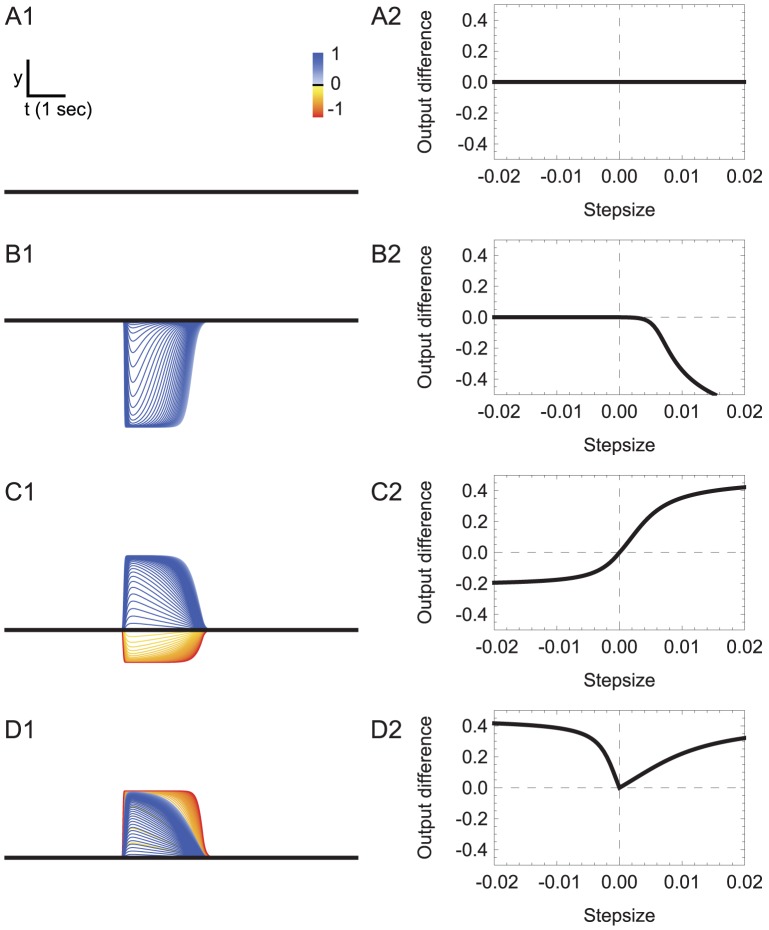
Interneuron types based on their response to step changes in concentration. Type **A**: insensitive. Type **B**: sensitive only to either upsteps or downsteps. Type **C**: ambiguously sensitive to both upsteps and downsteps. Type **D**: unambiguously sensitive to upsteps and downsteps. Figures in column **1** show neuron output trace over time, colored according to the different steps in concentration received. The sign and magnitude of the step in concentration is given by the color bar. The black trace represents the output of the neuron without any input. Column **2** shows their corresponding sensitivity plot. Average output of the neuron (relative to the average output of the neuron in the absence of any input) as a function of step changes in concentration of different sign and magnitude over the range of sensory stimuli. Flat regions of the curve correspond to areas of no-sensitivity to input over that region. The slope of the curve denotes the degree of sensitivity to changes around that region of the input.

Due to the nonlinearities of the input-output relation, interneurons can react to changes in concentration only over a limited range (e.g., Figure 8A). We identified four types of interneuron dynamics: insensitive (type *A*, Figure 10A), sensitive only to either upsteps or downsteps (type *B*, Figure 10B), ambiguously sensitive to both upsteps and downsteps (type *C*, Figure 10C), and unambiguously sensitive to upsteps and downsteps (type *D*, Figure 10D). There are two factors that determine the type of a cell. The first is the location of the bias with respect to the incoming weights. In cells of type *A*, the bias is saturating the neuron output and both incoming weights push in the direction of saturation. In cells of type *B*, the bias is in the saturated region, but the sensory neurons connect with opposite polarities and one of them drives the net input into the sensitive area of the sigmoid. In cells of type *C* and *D*, the bias is centered on ranges of the sensitivity. The second determining factor in the type of the cells is the polarity of the incoming weights from the ON and OFF cells. The incoming weights for type *C* cells have the same polarities, whereas the incoming connections in type *D* cells have opposite polarities.

#### AIY

In the high performance subpopulation, 6 of the 54 cells were type *A*, 10 were type *B*, 12 were type *C*, and 26 were type *D*. Klinotaxis networks have two AIY cells, a left and a right one. What matters to klinotaxis is the combination of the pair. The majority of successful networks (25 out of the 27) had a type *D* AIY cell paired with any type of other AIY cell, in roughly similar proportions: 6 were paired to a type *A*, 9 were paired to a type *B*, and 9 were paired to a type *C*. Only one of the networks has two type *D* cells. The remaining two networks have a combination of types *C* and *B*.

How is information about the chemical concentration distributed across the pair of AIY cells? In order to determine how much of the information about the stimulus is redundant or complementary in the left and right cells, we compared the range over which the cell is sensitive to changes in sensory input (henceforth, coverage, see Methods), individually versus combined (Figure S2A). Across the successful population, individual AIY cells covered only a fraction of the range covered by two cells combined, on average 0.65. Therefore, although there is some overlap in the range of sensitivities, the left and right cells in all networks specialize their sensitivities to only a part of the sensory range. We can also ask how much information the two neurons are sharing. The only connection between the two neurons is via the gap junction. We studied the role of the gap junction by comparing the coverage of the AIY pair while blocking the gap junction between the left and right cells (Figure S3A). Across the successful population, the gap junction did not play an important role in the transformation that occurs in the AIY layer. That is, for the majority of networks in the successful population, blocking the AIY gap junction leaves the individual AIY cells with only a minor decrease in its coverage over the sensory input compared to the unblocked scenario.

**Figure 11:**
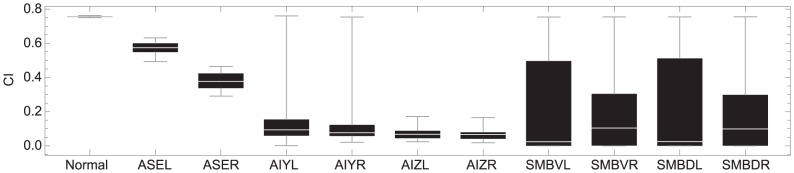
Effects of simulated neuron ablations on klinotaxis performance. Neuron activation set to resting potential (such that neuron output is 0) during assay. The middle bar inside the box is the statistical median. The outer edges of the box represent the 0.25 and 0.75 quantiles. The lines that extend out of the box span the full dataset.

Ablating the chemosensory neurons ASEL and ASER resulted in qualitatively similar deteriorations to klinotaxis performance to what has been observed experimentally [63] and in the previous model [77] (Figure 11). The current neuroanatomical model allowed us to also explore the effect of ablating individual interneurons, which had not been possible in previous models and has not yet been done in the worm (Figure 11). Ablating any one of the AIY cells individually decreased klinotaxis performance severely in most of the successful networks. Nevertheless, as can be seen by the variance, there were some networks in the high scoring subpopulation that performed klinotaxis even after ablating one of the AIY cells.

#### AIZ

**Figure 12:**
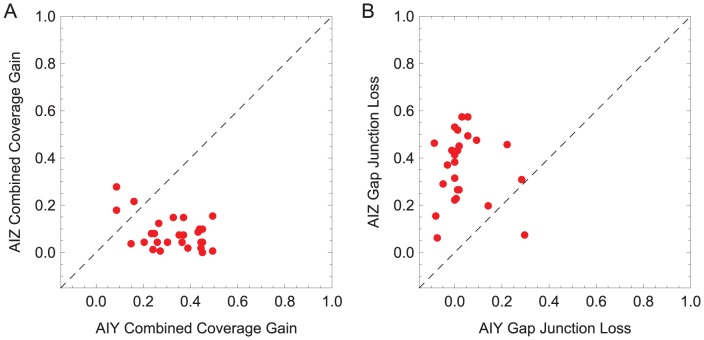
Interneuron comparison. **A**, Unique information about the sensory input in the interneurons. The coverage of a cell measures the range of the sensory stimuli over which it is sensitive to changes. When the left and right cells specialize over a different range of possible sensory stimuli, their combined coverage can be greater than their average independent coverage. The *x*-axis represents the gain in coverage when the left and right AIY cells are considered jointly from when each cells is considered independently. The *y*-axis represents the same information for the AIZ pair. **B**, Role of gap junctions in the interneurons. Effects of simulated blocked gap junction on coverage of the interneurons over the range of step changes in concentration for AIY and AIZ cells. The *x*-axis represents the loss in coverage when the AIY gap junction is blocked, compared to when it is functioning normally. The *y*-axis represents the same information for the AIZ pair. Each point represents a network from the successful population.

All cells in the second layer of interneurons were type *D* (Figure 10D). That is, each AIZ left and right cell is individually sensitive to upsteps and downsteps. This can be seen when we compare the coverage of individual AIZ cells with the combined coverage over the pair (Figure S2B). Across the successful population, the coverage of the individual AIZ cells was more similar to the range covered by two cells combined, on average 0.83. In the majority of the networks in the successful population (24 out of 27), the overlap between the shared coverage of the range of sensitivities in the left and right AIZ cells is greater than in the AIY cells (Figure 12A). Neuroanatomically, this is not intuitive because when we only consider the chemical synapses, the sensitivity of each AIZ cell is constrained by the sensitivity of the AIY cell upstream from it. The explanation for the increased shared coverage in the AIZ pair is to be found in the role of their gap junction (Figure S3B). When we compared the coverage of the AIZ neurons while blocking the gap junction with the normal coverage, we observed a larger detriment to the coverage of the sensory range with respect to the AIY neuron. The left and right AIZ cells mutually increase their sensitivity to a larger range of the spectrum via the gap junction. In the majority of successful networks (24 out of 27), the gap junction plays a more important role in the AIZ layer (by broadening the range of sensitivities) than in the AIY layer (Figure 12B). Finally, ablating any one of the AIZ cells individually decreased klinotaxis performance substantially in all of the successful networks. Unlike ablations to AIY, ablations to AIZ result in a decrease in klinotaxis performance across all high-performing individuals. This suggests that both AIZ cells are likely to be crucial for klinotaxis in the biological organism, but not necessarily both AIY cells.

#### SMB

Motor neurons all produced the same strategy: upsteps in concentration result in reduced difference between the dorsal and ventral motor neuron outputs and downsteps in concentration result in a larger difference between the dorsal and ventral motor neuron outputs. Although the specifics of how each circuit accomplishes this varies, three common features are observed. First, the sensitive part of the SSIO curve is biased to the side of the oscillatory range towards which the input changes during downsteps (to the left of the center of the oscillatory range when the input increases during upsteps and decreases during downsteps, and to the right of the oscillatory range when the input decreases during upsteps and increases during downsteps). The effect that downsteps have on the net input of the motor neurons is a function of the input/output relationship between changes in concentration and changes in output of AIZ (positive relationship, increase in concentration leads to increase in AIZ output and vice versa; negative relationship, increase in concentration leads to decrease in AIZ output) and the polarity of the AIZ-SMB connection. Second, in the majority of motor neurons the sensitive part of the SSIO curve falls within the bounds of the oscillatory range. This means that each motor neuron can react to upsteps and downsteps by increasing or decreasing the difference between the dorsal and ventral cells. Because there are two pairs of dorsal/ventral motor neurons, a left and right pair, in most successful networks one of the pair of motor neurons was more sensitive to upsteps (i.e., most of the sensitive region was within the oscillatory range) while the other pair was more sensitive to downsteps (i.e., most of the sensitive region is outside of the oscillatory range). Third, the SSIO curve of the majority of motor neurons was unistable (self-connection <4). Crucially, all solutions had at least one unistable pair of motor neurons. Therefore, motor neurons react smoothly to different changes in concentrations. In the few cases where the bistable motor neurons were found, the abrupt change was in reaction to large decreases in concentration. We also performed individual ablations on the motor neurons. We observed a wide variance in the decrease of performance when ablating individual motor neurons (Figure 11). No subsets of motor neurons were responsible for the modulation of the turning across the entire population. Furthermore, for the majority of the networks (18 out of 27), ablations to all four motor neurons affected klinotaxis substantially. For the rest of the networks, at least two of the motor neurons were crucial.

## Discussion

In this paper, we used evolutionary algorithms to set the unknown electrophysiological parameters of a minimal salt klinotaxis circuit extracted from the *C. elegans* connectome such that a simple brain-body-environment model of the worm exhibited efficient chemotaxis. We first analyzed in detail the operation of a high-performing solution and then explored the extent to which similar principles were operating throughout the ensemble of high-performing solutions. Due to the underconstrained nature of the problem, the particular parameter sets found by different runs of the evolutionary algorithm varied widely even when producing very similar chemotaxis behavior. However, several broad classes of patterns observed in the ensemble of high-performing solutions suggest new experiments that need to be carried out in order to select between these possibilities and further refine the model.

First, a more systematic analysis of turning as a function of the gradient in the translational direction in the worm is required. In our models, we observed larger magnitude turns for negative translational gradients than for positive translational gradients across all successful networks (Figure 4C). Although this was not a criteria set forth in our definition of klinotaxis, it is an efficient supplement to the strategy, exploited by the evolved model worms. Essentially, the magnitudes of the corrections are larger when the worm is heading away from the peak than when the worm is heading towards the peak. This has not yet been analyzed in the worm.

Second, ablations of individual AIY and AIZ cells (as opposed to both members of a class simultaneously) should be performed. The variance in klinotaxis performance for AIY-ablated model worms was higher than for AIZ-ablated model worms in the successful population. Our analysis revealed that the distribution of the information about the sensory stimuli was better distributed across AIZ cells and AIY cells, where sometimes all of the information resided in only one of the cells, and none of the information in the other one. This is influenced by the neuroanatomy of the circuit, where AIY cells have access to information from both the ON cell and the OFF cell, enabling an individual cell to integrate most of the necessary information to perform the task. AIZ cells, on the other hand, receive already integrated information from the AIY cells, and have to distribute the information to other AIZ cell, via the gap junction, in order to have an effect on all four motor neurons. So far klinotaxis experiments involving AIY and AIZ have been performed only while ablating the left and right cells simultaneously [63]. Unfortunately, simulated ablations of the whole class do not make sense in this minimal model because there are no alternative pathways, thus making it difficult to compare the existing experimental results to simulated ablations. Nevertheless, the results of the model are consistent with the ablation experiments in the real worm, where killing AIY also has higher variance than killing AIZ [63]. The variation, of course, could be attributed to different reasons in the model and the worm. The variance in the simulation data arises from the different parameters of each of the successful klinotaxis networks. There are at least two possible sources of variation in the worm data: experimental ‘noise’ created by variability in the observational and experimental techniques, and natural variability in the worms [31].

Third, a more refined set of ablation experiments could also test which pattern of AIY sensitivities that we observed in our model ensemble is utilized by *C. elegans*. Analysis of the dynamics of model AIY interneurons revealed three types of successful networks within the population: networks where only one AIY cell was sensitive to the full range of changes in concentration, networks where left and right cells had a different range of sensitivities to changes in concentration, and networks where both cells covered the full range of changes in concentration. Ablation experiments to individual AIY cells could distinguish between all three scenarios in the worm. In the first scenario, ablating one of the AIY cells would lead to a major decrease in performance, whereas ablating the other AIY cell would have no effect on performance. In the second scenario, killing either AIY cell individually would not decrease performance entirely, but the behavioral deficiency between ablating left and right cells would be significantly different. In the third scenario, the behavioral deficiency when ablating left and right cells would be similar. The majority of networks exhibit a different range of sensitivity over the sensory input in the left and right cells. This is similar to what has been observed in ASE [69]. Our analysis could help distinguish the AIY cells further. A study of the resulting behavioral pattern of left and right ablations could allow us to infer the difference in the range of sensitivities between the two cells. Networks without sensitivity to upsteps produce less efficient spiral tracks towards the peak, but just as reliably. On the other hand, networks without sensitivity to downsteps produce efficient paths straight towards the peak, but only for some orientations.

Fourth, any additional physiological analysis of the relationship between changes in concentration and interneuronal activity would help to narrow down the arithmetic sign possibilities in this circuit. From our analysis, we know the paths from ASEL to the motor neurons and from ASER to the motor neurons are antagonistic: an upstep in concentration increases the net input to the neck motor neurons, and a downstep in concentration decreases the net input, or vice versa. Crucially, we know from our analysis which of the two it ought to be based on how the sensitive region of the motor neurons is biased with respect to the oscillatory range. Essentially, the polarities of the connections must be such that an increase in concentration shifts the input towards the most saturated part of the range and a decrease in concentration shifts the input towards the sensitive part of the range. Because there are three chemical synapses between the chemosensory neuron and the motor neuron, there are a total of 8 possibilities. Of course, direct electrophysiological study of these connections in the worm would be ideal, but other experimental possibilities exist. For example, characterizing the sensitivity of the motor neurons with respect to the locomotory oscillation would help to narrow down the set of possible polarities that can result in successful klinotaxis to half.

Fifth, blocking individual gap junctions between the two interneuron classes, AIY and AIZ, would provide insight into the relationship between number of contacts and functionality. Our analysis revealed that the functional role of the AIZ gap junction was more crucial than the role of the AIY gap junction in the successful population. The anatomy of the chemical synapses in the network is likely to be playing a key role. AIY has access to the full range of input from the incoming chemical synapses of ASER and ASEL, whereas AIZ cells depend only on the nonlinear output of the AIY cells upstream from it. This was also reflected in the evolved strength of the gap junctions in the successful population, with a median ratio of 0.69 between the strength of the AIY/AIZ. This result is roughly consistent with the corresponding ratio derived from the known neuroanatomy in the worm: the AIY gap junction has one contact whereas the AIZ gap junction has two contacts [1].

Not all of the experiments we have proposed are equally feasible. The first experiment could be performed using detailed behavioral data from chemotaxis assays. Experiments 2 through 4 require individual cellular ablations that are currently possible. The last experiment, testing the role of the gap junction and the way in which information is shared in the interneurons, AIY and AIZ, is not currently feasible in *C. elegans*.

**Figure 13:**
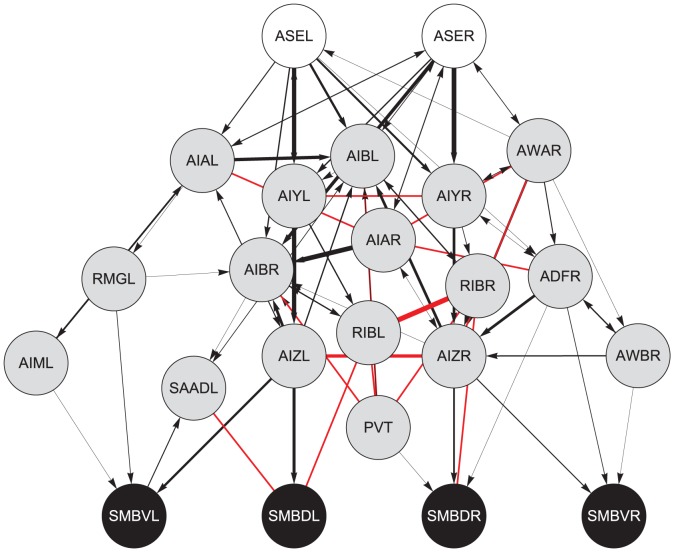
Neuroanatomical network connecting ASE to SMB, constrained to paths of length 3, but without a contact threshold. White disks, chemosensory neurons; gray disks, interneurons; dark gray disks, motor neurons. (Note: ADFR is a chemosensory neuron, but it is not shown in white because it is not in the original target set. Similarly, RMGL is a motor neuron, but it is not shown in black because it is not in the motor target set). Black connections represent chemical synapses; red connections represent electrical gap junctions.

It is important to reiterate that the current minimal model is not the ultimate model of the actual klinotaxis circuit, only a useful starting point for the modeling-experimental cycle. There are several directions in which our minimal klinotaxis model could be extended. First, as limitations of this minimal model are encountered, additional interneuronal pathways should be considered. For example, Figure 13 shows the circuit obtained by relaxing the contact threshold constraint in our *C. elegans* connectome search; it contains an additional 13 neurons and 18 feedforward paths between ASE and SMB. Similar extensions could be obtained by relaxing the path length constraint. Second, as additional experimental observations are made, neurons should be added or deleted from the network. For example, a recent study shows that RIA encodes head movement [83]. A subsequent model could explore the paths between ASE and the neck motor neurons, through RIA. Third, more biological realism can be introduced to the model as necessary to account for new experimental results. As more neurophysiological information becomes available about *C. elegans* neurons, neuromodulation, and the relationship between contact number and strength of interaction [48], [69], [84], [85], more biophysically-grounded neural models can be employed. In addition, more realistic models of the body musculature could be employed [86], [87]. Finally, a neuroanatomically-grounded model of *C. elegans* klinotaxis could serve as a springboard for other future modeling efforts, including the interaction of klinotaxis with klinokinesis [64], [71], its integration with locomotion [87], associative learning [66], [88], and its relationship with other taxes such as odortaxis and thermotaxis [78], [89]. In the long run, a model such as the one we have described here may represent an initial step along a path to the ultimate goal of having a brain-body-environment model of a complete animal.

In this paper, we have shown how a stochastic optimization technique such as evolutionary algorithms can be used as a kind of semi-automated hypothesis generator. By combining known neuroanatomical constraints from the *C. elegans* connectome with reasoned simplifications of its body and environment, optimization can fill in missing electrophysiological parameters in plausible ways so as to produce worm-like klinotaxis. Since our knowledge of any biological system is always partial, this methodology can be applied more generally: optimization can be used to explore the possibilities for what is unknown in ways consistent with what is known. A key feature of this approach is that the result is not a unique model, but rather an ensemble of models that are consistent with current knowledge of the system of interest. By studying the structure of this ensemble, one can formulate new experiments that can distinguish between the various classes of possibilities. The results of these experiments can then be used as additional constraints for subsequent optimizations in an iterative cycle of model refinement. In this way, productive interactions between modeling and experiment can begin very early in the lifecycle of a biological modeling project, when very little data is available, and carry through to a mature project when the system has been very well-characterized experimentally.

## Methods

Our model utilized the same chemosensory model, neuron model, chemical synapse model, and simplified head model as the previous klinotaxis model [77]. The primary difference was that the new model included interneuronal pathways derived from an analysis of the *C. elegans* connectome. In addition, the new model also included electrical synapses.

### Connectome data mining

Neuronal connectivity data for *C. elegans* was assembled by White et al., [1] from 5 animals, and later revisited [2], [24]. For each neuron, data exists for the total number of chemical synapses. There is also information about the synapse type: gap junction, where there is no directionality; an unambiguous chemical synapse from one neuron to another, also called a monadic synapse; and a joint chemical synapse between one neuron and more than one recipient, which can be dyadic or triadic. The *C. elegans* connectome data set is not 100% complete. Connectivity data for 39 of the 302 neurons is partially missing, including the most posterior 21 of the 75 motor neurons. Current theoretical and experimental studies are aimed at estimating and reconstructing missing data [90], [91]. The klinotaxis network focuses on neurons that are in the head and neck, which is where the data is most complete. While it is possible for the missing data to change the results, there is no reason to wait for its full reconstruction to begin to develop the methods of analysis to link the connectome to behavior.

In order to search the connectome, we developed code that finds paths connecting two sets of neurons in the *C. elegans* connectome. Using existing online tools (e.g., [92], [93]), it is possible to manually examine the connections between pairs of neurons. However, no tool was available to systematically search the connectome for all pathways connecting two sets of neurons that satisfy a flexible set of search criteria. Our code recursively performs a breadth-first search of the *C. elegans* connectome database from a *Root Set* of sensory neurons to a *Target Set* of motor neurons subject to a set of constraints. At each step of the search, two constraints were applied. A *Depth Limit* constraint terminated the search at a specified pathway length. A *Contact Threshold* constraint only considered connections that involved more than a given number of chemical synapses or gap junctions.

### Sensory neurons

Changes in salt concentration were encoded by ON and OFF chemosensory cells [69] using an instantaneous function of a derivative operator applied to the recent history of attractant concentration [94]:
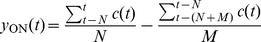
(1)where *c(t)* is the concentration at time *t*, and *N* and *M* are the durations of the two intervals over which the concentration is averaged. In response to a concentration step of infinite duration at 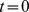, 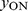 yields a linear rise to a peak at 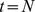, and a linear decay to base line at 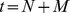; accordingly, *N* and *M* are referred to as the “rise time” and the “decay time” of the sensory neurons. In the case of the OFF cell, 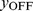, the signs were inverted so that decreases in concentration yielded positive activations. In both ON cells and OFF cells negative activations were set to zero.

### Interneurons

Interneurons were modeled as passive, isopotential nodes according to:
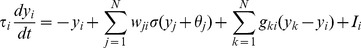
(2)where *y* represents the membrane potential (or neuron activation) relative to the resting potential (thus *y* can assume positive and negative values), 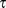 is the time-constant, the first sum term is the input from the chemical synapses, the second sum term is the input from the electrical synapses, and the third term represents external input to the neuron.

The model assumed chemical synapses release neurotransmitter tonically and that steady-state postsynaptic voltage is a sigmoidal function of presynaptic voltage [56]:
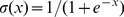
(3)where 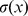 is the synaptic potential or output of the neuron. The chemical synapse has two parameters: 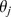 is a bias term that shifts the range of sensitivity of the output function, and 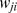 represents the strength of the chemical synapse. We can interpret the strength as the product of the number and size of the chemical synapses.

The importance of electrical synapses has been shown in several *C. elegans* behaviors, including locomotion and touch-withdrawal behaviors [95], [96]. Electrical synapses are generally described as rectifying (current passes preferentially in one direction) or non-rectifying (current is passed equally efficiently in both directions). Unfortunately, there is no concrete evidence about the nature of electrical synapses in *C. elegans*. Until more evidence is available, and in line with previous models [97], the model assumes electrical synapses in *C. elegans* are nonrectifying, with 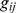 as a conduct conductance between cell *i* and *j* (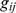
>0).

### Neck motor neurons

Neck motor neurons were modeled similar to the interneurons, except with self-connections. Biophysically, self-connections can be interpreted as the voltage dependence of inward currents underlying the graded regenerative potentials that are characteristic of several *C. elegans* neurons, including the neck motor neurons [48], [84]. The neck motor neurons also receive an additional input from an oscillatory component, 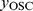.
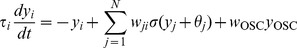
(4)where 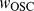 represents the strength of the connection from the oscillatory component. Because the cellular mechanism by which oscillations are generated during locomotion in *C. elegans* is unclear, we did not explicitly model this mechanism; instead, we represented its effect as a sine wave, 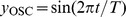, with *T* = 4.2 sec, the duration of a one cycle of locomotion on agar [73]. The dorsal and ventral motor neurons receive out of phase input from the oscillatory component.

### Body and behavior

**Figure 14:**
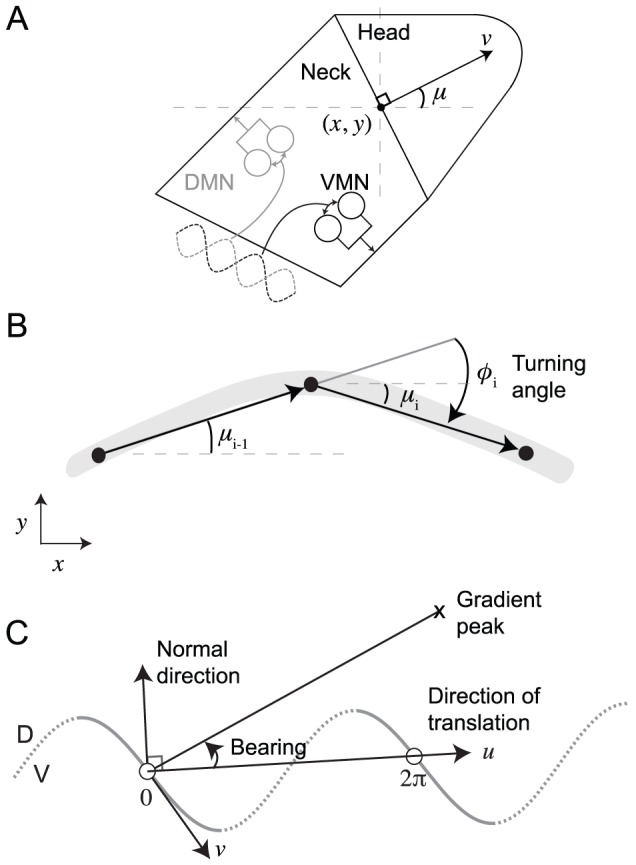
Model worm. **A**, Idealized body. The model worm was represented as a point (*x*, *y*) located at the center of the border between idealized head and neck regions of the model. The quantity *μ* is the angle between the instantaneous velocity vector *v* and the positive *x* axis. The dorsal (gray) and ventral (black) motor neuron pairs receive out of phase oscillatory input from the body. **B**, Changes in orientation. Between time steps *i*−1 and *i*, the orientation of the velocity vector changes by the turning angle *φ*. The gray arc is the model worm's path. **C**, Terminology. Orientation vectors used in the analysis of sinusoidal locomotion. Undulations occur in the *x–y* plane. The *white circles* represent the start and end of one locomotion cycle. Solid section of the trajectory represents ventral head sweeps. Dashed section of the trajectory represents dorsal head sweeps.

In sinusoidal locomotion (without slip), each body segment follows the one anterior to it. The worm was therefore represented as a single point (*x*, *y*) with instantaneous velocity *v*. The angular direction of movement μ was measured relative to the positive *x*-axis (Figure 14A). The biomechanics of locomotion were represented in idealized fashion, with two main assumptions. (1) Neck muscle length was proportional to motor neuron output. (2) The turning angle 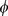 (Figure 14B) was proportional to the difference in muscle length. After combining constants of proportionality, this gives:
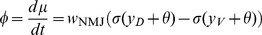
(5)where, 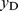 and 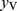 are activations of the dorsal and ventral neck motor neurons, 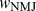 is the strength of the connection from motor neurons to muscles. It follows that the model worm's position is updated as:
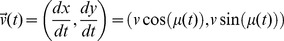
(6)where *v* is a constant speed of 0.022 cm/s [73]. To include pirouettes, the model worm's orientation was randomized with an average frequency of 0.033 Hz, which matches the baseline frequency of pirouettes in real worms [71]. In analysis, pirouette frequency was set to zero.

We did not explicitly model the mechanism responsible for generating the oscillations for forward thrust; instead, we represented its effect as a sine wave. Movement in real worms cannot occur without the thrust generated by undulations [98]; to implement this constraint, the velocity of the model worm was set to zero unless undulations were present.

### Environment

The gradient during a typical salt chemotaxis assay has a Gaussian shape [64]. In the context of evolution, however, Gaussian gradients are problematic because local steepness is systematically related to distance from the gradient peak. To avoid this problem, we used conical gradients of varying steepness during evolution. Accordingly, attractant concentration 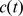 was proportional to the Euclidean distance from the gradient peak,
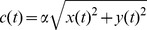
(7)where 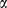 determines the steepness of the gradient.

### Evolutionary algorithm

The parameters of the model were evolved using a genetic algorithm [99]. The optimization algorithm was run for populations of 60 individuals. We evolved the following parameters (ranges are shown in brackets): 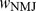 [1, 3]; 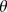, 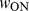, 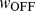, and 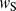 [−15, 15]; 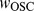 [0, 15], *N* and *M* [0.1, 4.2]. Network parameters were symmetrical across the dorsal/ventral midline. Parameters were encoded in a 20-element vector of real-values between [−1, 1]; when needed, parameters were linearly mapped to their corresponding ranges. Each time the algorithm was run, individuals were initialized by random selection from the range of each parameter. Populations were evolved for 300 generations. At the end of a run, the parameters of the best performing individual were stored for later analysis. The algorithm was run 100 times (using different random seeds), yielding 100 distinct networks.

### Evaluation of fitness

Fitness was evaluated in simulated chemotaxis assays. At the start of each assay, the model worm was placed with a random orientation at a point 4.5 cm from the peak of the gradient and motor neuron activations were randomized over the range [0, 1]. Gradient steepness α was randomized over the range [−0.38, −0.01]. The fitness score was quantified in terms of a chemotaxis index CI defined as the time average of the distance to the peak of the gradient,
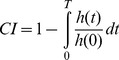
(8)where *h*(*t*) is the Euclidean distance to the peak, *h*(0) is the model worm's initial distance from the peak (4.5 cm), and *T* is the total simulated assay time (500 sec). For simplicity, negative CI values were set to zero. The fitness of an individual was defined as the average CI over 50 assays.

### Terminology

A common measure of chemotaxis performance in *C. elegans* salt chemotaxis assays is the proportion of worms that reach the gradient peak [35], which we will refer to as *reliability*. In our simulations, we defined the peak to be a region enclosed by a circle with a radius of 0.1 cm centered on the peak.

A locomotion cycle consists of alternating ventral (solid trajectory, Figure 14C) and dorsal (dashed trajectory, Figure 14C) *head sweeps*. The principal orientation vector used was the *direction of translation*, defined by any pair of points on a trajectory separated by a phase difference of 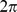, i.e. one cycle of locomotion (Figure 14C). The vector 90 degrees counter-clockwise from the direction of translation is the *normal direction*, which was used to quantify the gradient as sensed by the model worm over a single head sweep. The angle between the line of steepest ascent and the direction of translation is the model worm's *bearing*. The *turning bias* was defined as the sum of the turning angle 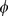 over one cycle of locomotion.

The evolved model neurons are sensitive to sensory input over a certain range. The *coverage* of a neuron was defined as the proportion of sensory input, over a specified range, where the output of the model neuron was substantially different from the output of the neuron over nearby stimuli. The range was determined by the sensory input observed during a usual klinotaxis run, ±0.02. The average output of the neuron was recorded for different steps in concentration over that range, in intervals of 5×10^−4^. Each point was considered ‘covered’ only if the average output of the neuron for that input was sufficiently different (greater than 1×10^−4^) than the average output during the previous step in concentration.

## Supporting Information

Figure S1 Parameter study for the best evolved network. We tested the chemotaxis performance of the representative network as individual parameters were modified between ±10% of their original value. On the *y*-axis is the chemotaxis index of the network (shown between 0.75, the cutoff point used for the successful population, and 0.89). The gray lines show the degradation of the network's performance as individual parameters are independently perturbed away from the evolved values. The thick black line shows the average degradation over all parameters in the network. As expected, the network's performance is more sensitive to some parameters than others. The most sensitive parameters were the self-weights and biases on the motor neurons, and the weights from AIZ to SMB. All parameters exhibit a graceful degradation over a larger range of perturbations (0%–200%, points not shown).
(EPS)

Figure S2 Combined versus independent coverage across interneuron pairs. Coverage of interneurons AIY (**A**) and AIZ (**B**) over the range of step changes in concentration when the left and right cells are considered independently versus combined. Data shown for all individuals in the successful population. Gray points represent the coverage of the left and right cells individually, joined by a line to identify the network. Red points represent the average coverage for that network in the two conditions: individually and combined.
(EPS)

Figure S3 Role of gap junctions in the interneurons. Effects of simulated blocked gap junction on coverage of the interneurons over the range of step changes in concentration for AIY (**A**) and AIZ (**B**) cells. Data shown for all individuals in the successful population. Gray points represent the coverage of the left and right cells individually, when the gap junction is functioning versus their coverage when the gap junction is blocked. The data points for the left and right cells are joined by a line to identify the network. Red points represent the average coverage for that network in the two conditions: normal and blocked.
(EPS)
